# Effect of a Medication Adherence Education Intervention on Clinical Outcomes in Adults with Type 2 Diabetes Mellitus: A Randomized Controlled Trial Study

**DOI:** 10.2174/0118715303335172241216102015

**Published:** 2025-01-17

**Authors:** Ali Tarighat Esfanjani, Maryam Zare, Afrouz Mardi, Arezoo Haghighian Roudsari, Maryam Rafraf, Manouchehr Iranparvar-Alamdari, Daniel Hackett, Abdolreza Shaghaghi, Mohammad Asghari Jafarabadi

**Affiliations:** 1 Nutrition Research Center, Faculty of Nutrition and Food Sciences, Tabriz University of Medical Sciences, Tabriz, Iran;; 2 Department of Nutrition, Khalkhal University of Medical Sciences, Khalkhal, Iran;; 3 School of Health, Ardabil University of Medical Sciences, Ardebil, Iran;; 4 Department of Community Nutrition, Faculty of Nutrition Sciences and Food Technology, National Nutrition and FoodTechnology Research Institute; Shahid Behshti University of Medical Sciences, Tehran, Iran;; 5 Nutrition Research Center; Faculty of Nutrition and Food Sciences, Tabriz University of Medical Sciences, Tabriz, Iran;; 6 Department of Internal Medicine, Faculty of Medicine, Ardabil University of Medical Science, Ardabil, Iran;; 7 Physical Activity, Lifestyle, Ageing and Wellbeing Faculty Research Group, School of Health Sciences, Faculty of Medicine and Health, The University of Sydney, Lidcombe, NSW, Australia;; 8 Department of Health Education and Promotion, Faculty of Health, Tabriz University of Medical Sciences, Tabriz, Iran;; 9 Cabrini Research, Cabrini Health, VIC 3144, Malvern, Australia; School of Public Health and Preventive Medicine, Faculty of Medicine, Nursing and Health Sciences, Monash University, VIC 3800, Melbourne, Australia;; 10 Road Traffic Injury Research Center, Faculty of Health, Tabriz University of Medical Sciences, Tabriz, Iran

**Keywords:** Diabetes mellitus, anti-diabetic drugs, glycemic control, transtheoretical model, medication adherence, HbA1C

## Abstract

**Background:**

Low adherence to Oral Antidiabetic Drugs (OADs) in adults with Type 2 Diabetes Mellitus (T2DM) leads to complications, death, and increased healthcare costs.

**Objective:**

This study aimed to evaluate the effectiveness of medication adherence education interventions for the clinical outcomes of adults with T2DM.

**Materials and Methods:**

Seventy adults with T2DM from an outpatient clinic in the City of Ardabil, Iran, participated in this study. The participants were randomized into an intervention group (*n*=35) or a non-interventional group (*n*=35). The intervention group underwent four educational sessions focused on adherence to OADs. Content within classes was influenced by a participant’s Stage of Change (SOC) result, which was related to behavior change modeling. Participants in the non-interventional group were placed on a waiting list to receive educational content after the follow-up. Assessments were conducted at baseline, four weeks, and six months.

**Results:**

Significant improvements in Fasting Plasma Glucose (FPG) (*P* = 0.018) and 2-hour Plasma Glucose (2-hPG) (*P* < 0.001) levels were found in the intervention group compared to the non-interventional group post-intervention. Additionally, HbA1C was significantly lower at follow-up in the intervention group than in the non-intervention group (*P* < 0.001). The Homeostasis Model of Insulin Resistance Assessment (HOMA-IR) revealed a significant increase in target cell sensitivity to insulin in the intervention group compared to that in the non-interventional during the follow-up period (*P* = 0.009). The SOC for adherence to OADs was significantly improved in the intervention group compared to that in the non-intervention group at the follow-up timepoint (*P*< 0.001).

**Conclusion:**

Medication adherence education interventions may improve the clinical outcomes of adults with T2DM.

**Clinical Trial Registration Number:**

IRCT201701165670N23.

## 1 INTRODUCTION

T2DM is a metabolic disorder characterized by elevated blood glucose levels, which may be due to impairment in insulin secretory function, Insulin Resistance (IR), and poor insulin secretion. This chronic disease is often accompanied by complications, such as damage to the heart and vasculature, kidney disease, blindness, peripheral nerve problems, retinopathy and neuropathy, dental problems, and amputation. It increases the risk of early death [1-3]. In 2019, T2DM affected 460 million adults and the number is expected to grow to 700 million by 2045 [4]. T2DM accounts for approximately 90-95% of all diagnosed cases of diabetes [5]. The Iranian population is susceptible to increased IR, and the prevalence of diabetes in Iran has been shown to be 11.3-14.6% [6]. For people with T2DM, insulin resistance forces β-cells to produce more insulin, with progression leading to the deterioration of β-cell functions and the inability to produce insulin [7]. Unfortunately, treatment cannot reverse the ongoing failure of β-cell function to prevent uncontrolled hyperglycemia and complications of diabetes [8]. HOMA-IR measures insulin sensitivity based on FPG and insulin concentration and is considered the gold standard in field investigations [9, 10]. Currently, there are no treatments to reverse the deterioration of β-cell function to prevent uncontrolled hyperglycemia and diabetes-related complications [7]. However, HOMA-IR is widely used in clinical practice to assess β-cell function and insulin resistance, and is estimated from fasting glucose and fasting insulin levels [11].

In addition to lifestyle modifications, most T2DM patients need medication adherence to achieve adequate glycemic control [12]. Pharmacotherapy plays a fundamental role in controlling blood glucose levels in patients with T2DM and improving health outcomes [13, 14]. There is a wide range of OADs available; however, the proportion of patients who achieve glycemic goals is low [15]. A potential factor for these low rates of attaining glycemic goals could be poor adherence to pharmacotherapy [16]. A strategy for addressing this problem in T2DM patients could be through education programs focused on increasing knowledge about available treatments [12, 17, 18]. Education empowers T2DM patients in terms of treatment and self-care [17]. However, it should be acknowledged that the use of OADs is accompanied by complex behaviors and barriers [17-20]. Therefore, careful selection of educational strategies and motivational techniques is required for behavioral change and overcoming barriers to self-management. A commonly used method for behavior change modeling is the Transtheoretical Model (TTM), which may enhance a T2DM patient’s motivation for self-management [21]. This model comprises four constructs: SOC, processes of change, decisional balance, and self-efficacy [22]. SOC involves five stages: pre-contemplation, contemplation, preparation, action, and maintenance. At each stage, people experience different changes and may have desirable progress for longer than six months [23]. T2DM patients in the pre-contemplation stage could show no intention to adhere to the OADs plan within the next six months. If they are in the contemplation phase, they may intend to adhere to OADs within the next six months. For T2DM patients in the preparation phase, there is an indication that they will adhere to OADs within the next 30 days. Finally, for a T2DM patient in the action stage, there is evidence of a change in their behavior (*i.e.*, following adherence to the OADs plan). In the last stage of the change process, adherence to OADs needs to be consistent for over six months [22].

Few studies have applied SOC to adults with T2DM in the field of self-care [24, 25]. However, there is a paucity of research that has investigated whether an education intervention with consideration of SOC is efficacious for improving self-management activities related to medication use in patients with T2DM [26]. Therefore, this study aimed to evaluate the effectiveness of a medication adherence education intervention for clinical outcomes in adults with T2DM from an outpatient clinic in Iran.

## 2 MATERIALS AND METHODS

### 2.1 Study Design and Participants

**Fig. (1):**
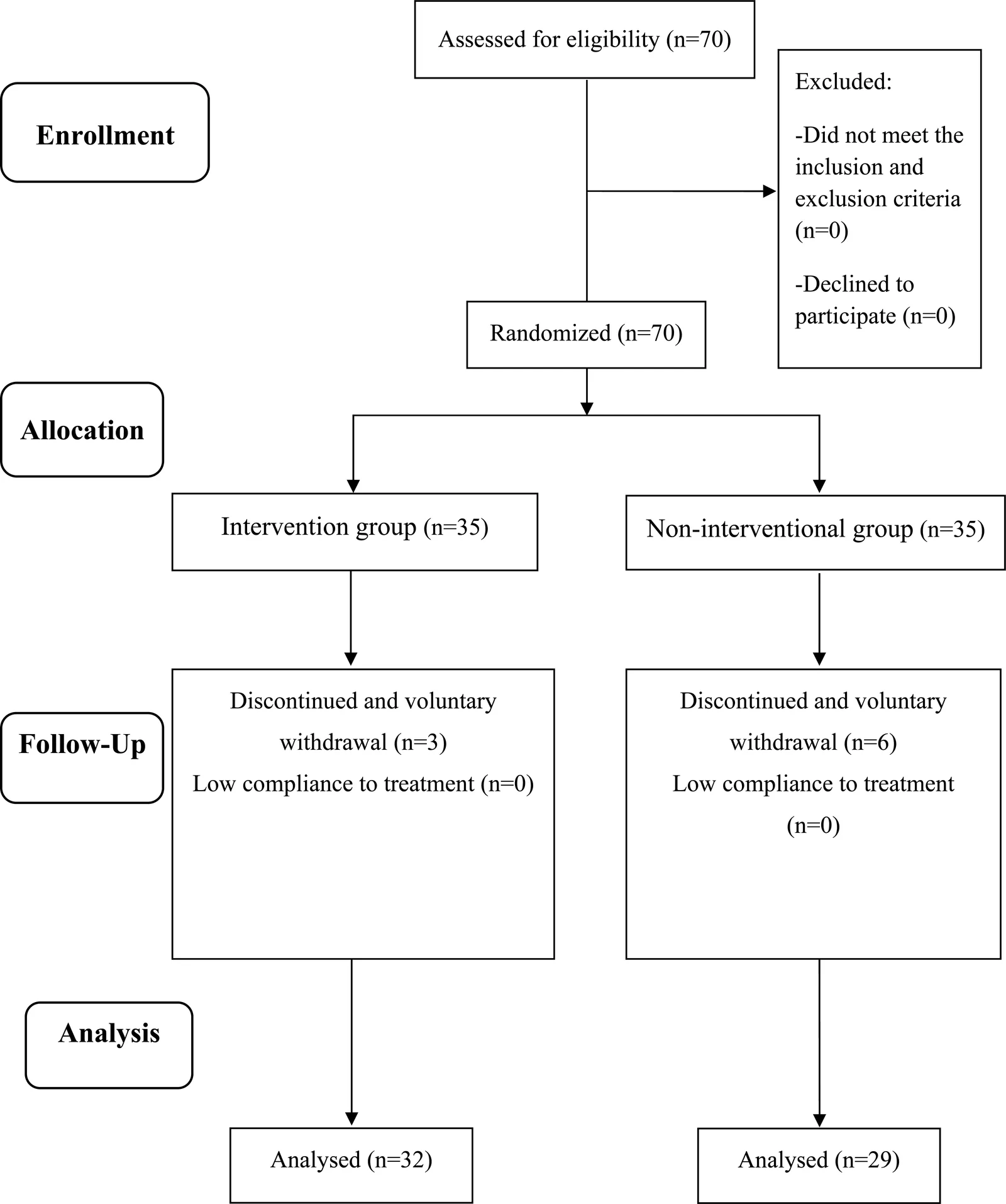
Flow diagram for participants included in the study.

This study was performed as a Randomized Controlled Trial (RCT). Seventy adults (37 females and 33 males) with T2DM were recruited by non-probabilistic convenience sampling from an outpatient clinic in the City of Ardabil. Participants were stratified by age and SOC and were single-blind randomized into two groups: an intervention group (*n*=35) and a non-interventional group (*n*=35). Randomization was performed using Random Allocation Software (RAS) after baseline testing (Fig. **1**). To be eligible for the study, potential participants were required to be aged ≥ 30 years and literate. The exclusion criteria included patients with comorbidities, such as cardiovascular disease, mental illness, insulin use, pregnancy, and lactation (females). A priori analysis was performed to calculate the required sample size (*n*=70) to reach the desired level of power. However, 9 adults were excluded from the analysis because they voluntarily withdrew from the study. The study was conducted using a single-blind evaluation.

### 2.2 Intervention

This study encompassed three distinct time points: baseline, post-intervention (four weeks), and follow-up (six months). Based on the results of the SOC questionnaire [27], the subjects were classified into four groups (pre-contemplation, contemplation, preparation, action, and maintenance) (Supplementary Material **S1**). The educational needs of the intervention group were determined according to the stage at which they were subjects, and there were SOC and different intervention strategies for moving the person to the next stage of change. Therefore, the class educational content was designed with content that was individualized to the needs of participants based on SOC results. After completing each stage of the training course, participants were transferred to the next stage. The educational content of the sessions is shown in Box 1. Participants were required to attend four 2-hour sessions over four weeks (*i.e.*, one session per week). Adherence to the OADs education program was designed based on participants’ needs in the SOC model. The educational sessions focused on adherence to OADs involved direct teaching methods, such as presentations using Microsoft PowerPoint, questions and answers, slideshows, and video projectors by a diabetes educator and nutritionist university professors. At the end of the classes, information booklets were distributed to the participants in the intervention group. The participants in the non-interventional group did not receive any form of education during the intervention. After the assessments at baseline, post-intervention, and follow-up, non-interventional subjects were offered the intervention. During the study period, all participants were requested to maintain their habitual dietary practices and physical activity levels. No adverse events or challenges were encountered during the intervention.

### 2.3 Data Collection

A series of structured questionnaires were used to collect information about participant characteristics, health and clinical status, and adherence to OADs. The sociodemographic characteristic questionnaire captured the participants’ age, sex, education level, occupation, and monthly income. For the health and clinical status questionnaire, information gathered included years since being diagnosed with T2DM, type of OADs, and number of OADs per day. Adherence to OADs based on SOC was measured utilizing the SOC in the self-management of patients with T2DM questionnaire, Iran **(**QSOCSMD-I). This questionnaire included three sections, including adherence to OADs, healthy diet, and physical activity, and comprised ten items. In adherence to the OADs section of the questionnaire, the first, second, third, fourth, and fifth multiple-choice questions referred to pre-contemplation, contemplation, preparation, action, and maintenance, respectively. The overall score was the sum of all five choices, ranging from 1 to 5 [27].

Blood samples were drawn and transfused into tubes containing EDTA in the morning after the participants had fasted for 12 h, and 2-hPG was measured 2 h after consuming breakfast. FPG and 2-h PG levels were measured using an enzymatic method and colorimetric analysis (GOD-PAP). Fasting serum insulin levels were measured using a chemiluminescent immunoassay (antibody or ELISA: ADVIA Centaur Insulin (IRI) antibody, human insulin; supplier: Siemens, catalog no. 02230141, RRID: AB_2909499, Germany). Insulin resistance was estimated as HOMA-IR = (FPG × fasting insulin)/22.5 in molar units [28]. All measurements were taken at baseline, four weeks, and six months post-intervention in both groups.

### 2.4 Statistical Analysis

The number of subjects in the intervention and non-intervention groups was calculated based on this formula. With regard to 80% capability at 95% significance, assuming an SD of 2% and HbA1C of 1% in the groups, and adjustment for a 10% dropout rate, a sample size of 70 was required for this research work. Data are presented as the mean ± standard deviation and frequency (percentage) for quantitative and qualitative variables, respectively. The Kolmogorov-Smirnov test was used to assess the normality of data distribution. Based on the normality test results, either an independent sample t-test or Mann-Whitney U test was used to compare the mean values of quantitative variables between the groups. The Friedman test and chi-square or Fischer's exact tests were used to evaluate the association between qualitative factors. Repeated measures ANOVA was used to compare results at all time points between the groups. In all statistical analyses, a *P*-value < 0.05 was considered statistically significant. Statistical analyses were performed using the SPSS software (version 23).

## 3 RESULTS

**Fig. (2):**
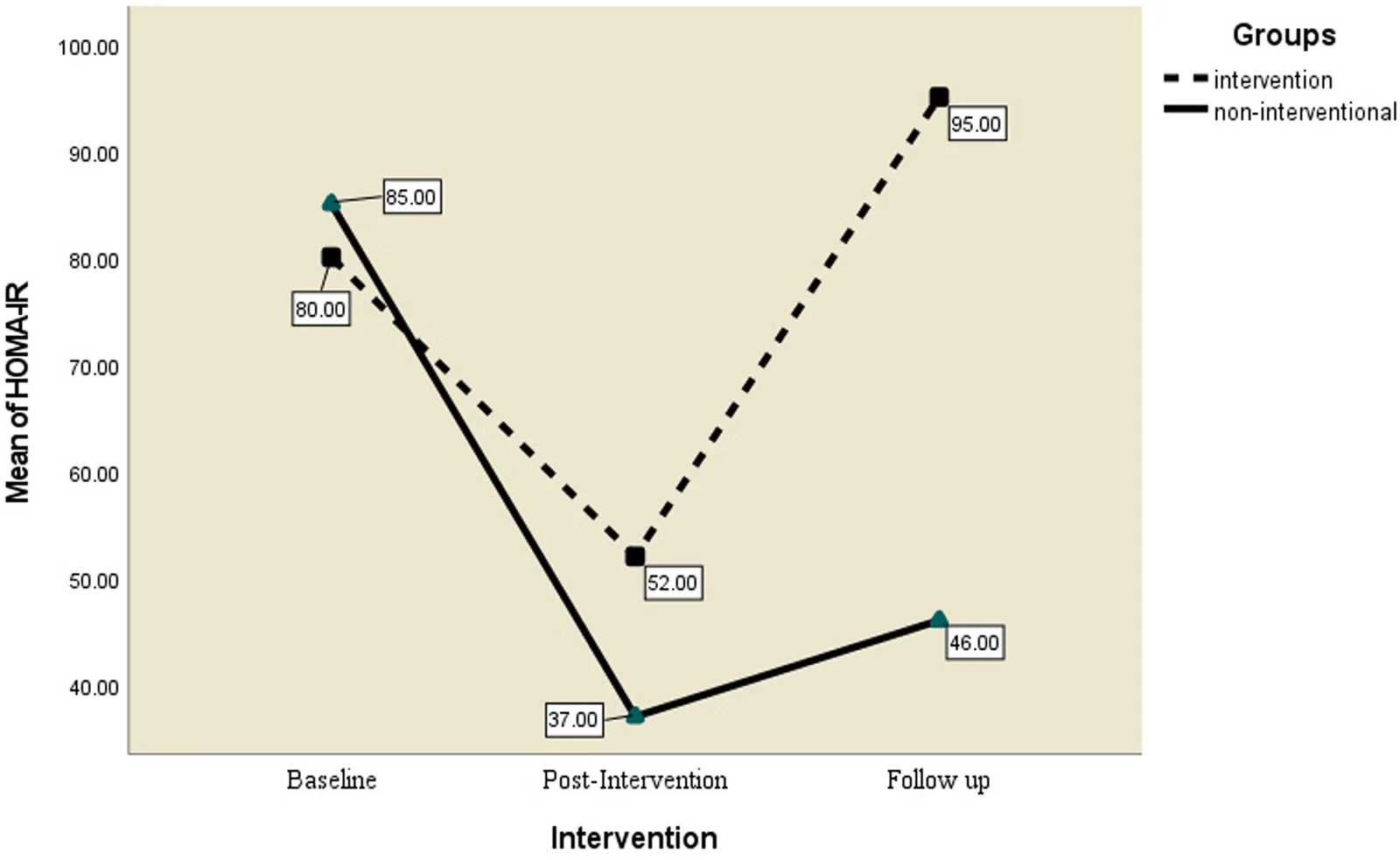
Mean Levels of HOMA-IR in Subjects at Baseline, Post-intervention, and Follow up. Repeated Measures ANOVA Adjustment for multiple comparisons: Sidak, *p > 0.05. Values are means ± SEM. HOMA-IR, Homeostasis model assessment of insulin resistance.

The mean age of participants in the intervention and non-intervention groups was 50.6 ± 7.2 and 52 ± 7.1 years, respectively. More participants in the intervention group (72%) than in the non-intervention group (28%) had a family history of T2DM (*P* = 0.033) (Table **1**). There were no significant differences between the groups at baseline for FPG, 2-h PG, fasting insulin, HbA1C, number of OADs, and HOMA-IR. A significant time × group interaction was observed for FPG levels (*P*= 0.015). Post-hoc analysis revealed FPG to be significantly lower post-intervention in the intervention group than in the non-intervention group (*P* = 0.018). However, there was no significant difference between the groups at follow-up (*P* = 0.117). Regarding 2-h PG, a significant time × group interaction was found (*P* = 0.035). The post-hoc analysis revealed 2-h PG to be significantly lower post-intervention in the intervention group than in the non-intervention group (*P* < 0.001), but no difference between groups was observed at follow-up (*P*= 0.113). There was no significant difference between the groups in terms of fasting insulin levels (*P*=.111). Another significant time × group interaction effect was found for HbA1C. The post-hoc analysis revealed that HbA1C was significantly lower at follow-up in the intervention group than in the non-intervention group (*P* = 0.016), but no differences between groups were found post-intervention (Table **2**). Post-intervention, there was no difference between the groups for HOMA-IR; however, the intervention group had a greater value than the non-interventional group at the follow-up timepoint (*P* = 0.009) (Fig. **2**).

The number of OADs used was not significantly different between the groups (*p* = 0.634) or throughout the study (*P* = 0.400). The types of OADs included metformin, Sulfonylureas (SU), and a combination of metformin + SU, which differed significantly between the groups at all time points (*P*<.001) (Table **3**). However, results from the Friedman test indicated no difference between participants in each group for the type of OADs used (*P* = 0.819). The results presented in Table (**4**) show that the SOC for adherence to OADs improved significantly in the intervention group compared to that in the non-intervention group at the follow-up time point (*P*< 0.001).

## 4 DISCUSSION

This study aimed to evaluate the effectiveness of medication adherence education interventions for clinical outcomes in adults with T2DM. The findings of the present study demonstrated the effectiveness of an educational intervention based on the SOC of the TTM model. The intervention group had significantly improved FPG, 2-h PG, HbA1C, and HOMA-IR levels after the intervention.

Basavareddy *et al.* conducted a cross-sectional study to assess the knowledge and attitudes of 150 doctors regarding prediabetes treatment. Most doctors considered diet and exercise as the first treatment if they recommended OADs in combination with diet and exercise [29]. Folse *et al.* conducted a cohort study to estimate the potential long-term consequences of clinical inertia, defined as the failure to start therapy or its intensification/non-intensification when appropriate, and found that adherence to OADs led to greater reductions in HbA1c and a lower risk of complications [30]. The present study’s findings were consistent with the results of Folse *et al.* in terms of HbA1c. Because the current study had only a 4-week intervention and a follow-up of 6 months, long-term complications/adverse events could not be investigated. Zare *et al.* explored the perceived facilitators and barriers of self-management in adults with T2DM based on the stages of the behavior-change model. This study found that physical-intellectual factors and socioeconomic situations negatively influence adherence to OADs. Adherence to OADs is facilitated by satisfaction with treatment and planning [22]. In the present study, the educational content of classes was focused on improving adherence to OADs in T2DM patients. As supported by our results, the educational intervention was effective in improving the clinical outcomes of the participants. These results were in agreement with a previous study that found that self-management education programs improve adherence to OADs in T2DM patients [20]. In contrast, several studies have found a self-management educational plan for medication adherence as ineffective in improving clinical outcomes in T2DM patients [27, 31, 32]. Additionally, another study showed that interventional education on self-care behaviors did not positively affect clinical outcomes in T2DM patients [33].

The number of OADs taken by participants in the present study did not differ between the groups, nor did it change over time. The increased number and percentage of adults with T2DM in the stage of action and maintenance (SOC) for OAD use after the intervention and at the 6- month follow-up provided an explanation for the success of the educational intervention. The results showed that the number and type of drugs used were based on the prescription of the treating physician based on the status of each adult with T2DM and that people with T2DM adhered to the prescribed number and type of drug according to the doctor's prescription. However, there was a difference between the groups in the types of OADs used. OADs work via four mechanisms: increasing the release of endogenous insulin and reducing FPG, affecting gastrointestinal absorption and renal reabsorption of glucose, changing site sensitivity to endogenous insulin, reducing gluconeogenesis/glycogenolysis or metabolism with medications, and use of medications acting on the incretin pathway (Glucagon-like Peptide-1 (GLP-1) analogs and Dipeptidyl Peptidase 4 (DPP4) inhibitors). These mechanisms of OADs result in increased insulin secretion and decreased blood glucose [34]. Several studies have demonstrated that changes in behavior may occur from two to eight months [35-38].

The results of the present study revealed that an educational intervention based on TTM can improve SOC for adherence to OADs, and improve FPG, 2h PG, and HOMA-IR at post-intervention, but in the follow-up, some results did not differ. Relapse is a common experience among subjects seeking to change health-related behaviors [39]. A relapse should not be viewed as a failure. It should be considered an excellent opportunity to reevaluate one’s triggers; reassess one’s motivation for change; reassess old/new barriers, including lack of programs, instructors, and coaches, problems with transportation, and lack of social support to achieve the goal; and plan for stronger contingency plans [40, 41].

To the best of our knowledge, this was the first study to examine the effectiveness of an educational program focusing on SOC to promote adherence to OADs. One main strength of this study was the use of an RCT design. Other strengths included using a variety of educational approaches, such as employing video projectors and PowerPoint, having participants talk about their experiences, the long follow-up (six months), completing the educational curriculum, and the use of the motivational TTM model. However, certain limitations should be noted. First, the sample size and follow-up duration were small. Second, the laboratory findings were based on reports from multiple local laboratories, which may have affected the accuracy of the data. Third, factors, such as a healthy diet and physical activity, were not measured in this study. Finally, our research enrolled a middle-aged and elderly population and a specific cultural and healthcare setting in Iran; therefore, this study’s findings did not represent the entire T2DM population.

## CONCLUSION

This study indicated a medication adherence education intervention based on SOC as effective in improving clinical outcomes (FPG, 2-h PG, HbA1C, and HOMA-IR) and adherence to OADs in patients with T2DM. It appears that SOC-guided educational programs may be a strategy to address the low rates of attaining glycemic goals in T2DM patients. The results of this study support the idea that medication adherence is a very important objective for attaining glycemic goals in the long-term self-management of patients with T2DM. Also, increased awareness of adherence to OADs has led to a reduction in the incidence of complications of chronic diabetes. In accordance with the TTM theory, relapse after intervention can be beyond social and demographic changes. Therefore, self-care interventions are important in the fields of motivation and psychology and may hold promise for minimizing relapse. This highlights the need for patient-centered medical care to include adherence to OADs in patients’ educational and counseling strategies. However, further research is required to corroborate these findings with larger sample sizes, longer educational interventions and follow-up durations, and a survey of lifestyle behaviors. It is also required to examine the effect of cultural variety on medication adherence to determine the sustainable impact of these approaches on health behavior change.

## Figures and Tables

**Table 1:** Demographic characteristic of participants in the Intervention and Non-interventional groups.

-		Intervention Group (n = 32)	Non-Interventional Group (n = 29)	*p*-Value
**Age (years)**		50.6 ± 7.2	52 ± 7.1	^†^0.473
**Age of onset diabetes**		43.9 ± 7.1	45.7 ± 8.8	^†^0.364
**Education level**	**Low**	19(59.4)	22(75.9)	‴0.220
	**High**	12(40.6)	7(24)	
**Family history**	**Yes**	23(71.9)	9(28.1)	*”0.033
	**No**	13(44.8)	16(55.2)	

**Note:** Data are expressed as mean ± SD or number (percentage). Percentages have been rounded and may not total 100. Analyses Analysis were^†^Independent Samples T Test, ”Mann-Whitney Test, Descriptive statistics, and ‴Chi-Square Tests.* *P*<0.05

Low: no education or primary and middle and under of high school diploma; High: Diploma and higher.

**Table 2:** Comparisons of the biomarker concentrations and number of taking oral anti-diabetic drugs (OADs) at pre, post-intervention and follow up between groups.

Variables	Time	**Group**		*p*-Value^a^		
		**Intervention Group (n = 32)**	**Non-Interventional Group (n = 29)**	Time	Group	Interaction
FPG-(mg/dl)	Baseline	202.97 ± 47.62	191.33 ± 57.652	<.001	.270	.015
	Post-intervention	155.45 ± 33.33	186.20 ± 63.91	-	-	-
	Follow up	148.96 ± 44.53	169.42 ± 69.61	-	-	-
2-h PG-(mg/dl)	Baseline	294.07 ± 79.43	312.37 ± 120.57	<.001	.042	.035
	Post-intervention	211.30 ± 57.43	284.20 ± 92.86	-	-	-
	Follow up	228.12 ± 67.02	252.67 ± 74.60	-	-	-
Fasting insulin-(mU/L)	Baseline	9.08 ± 11.03	9.87 ± 22.7	.131	.111	.142
	Post-intervention	7.94 ± 10.46	4.53 ± 2.90	-	-	-
	Follow up	14.62 ± 14.79	6.57 ± 6.25	-	-	-
HbA1C-(%)	Baseline	7.35 ± 1.25	7.37 ± 1.52	.929	.106	.016
	Post-intervention	7.10 ± 1.24	7.50 ± 1.44	-	-	-
	Follow up	6.81 ± 1.10	7.85 ± 1.36	-	-	-
The number of taking OADs	Baseline	3.28 ± 1.92	3.53 ± 1.89	.400	.634	.765
	Post-intervention	3.10 ± 1.67	3.36 ± 1.66			
	Follow up	3.28 ± 1.92	3.40 ± 1.93			

**Note:** Data are presented as mean ± SD. Analysis was ^a^ Repeated Measures ANOVA.

FPG, Fasting plasma glucose; 2-h PG, 2 hrs plasma glucose; OADs: oral anti-diabetic drugs

**Table 3:** Comparisons type of oral anti-diabetic drugs (OADs) during the study.

Time	Type of OADs	**Group**		*p*-Value
		**Intervention Group (n = 32)**	**Non-Interventional Group (n = 29)**	
Baseline	0	2 (6.3)	2(6.9)	<.001
	metformin	12(37.5)	6(20.7)	-
	glybiinclamid	17(53.1)	0(0.0)	-
	glyclazide	1(3.1)	2(6.9)	-
	Metformin+glybiinclamid	0(0.0)	17(58.6)	-
	Acrobaz+Metformin+glybiinclamid	0(0.0)	2(6.9)	-
Post-intervention	0	1(3.1)	0(0.0)	<.001
	metformin	15(46.9)	10(34.5)	-
	glibinclamid	15(46.9)	0(0.0)	-
	gliclazid	1(3.1)	1(3.4)	-
	Metformin+glybiinclamid	0(0.0)	16(55.2)	-
	Acrobaz+Metformin+glybiinclamid	0(0.0)	2(6.9)	-
Follow up	.0	1(3.1)	1(3.4)	<.001
	metformin	15(46.9)	7(24.1)	-
	glibinclamid	15(46.9)	2(6.9)	-
	gliclazid	1(3.1)	1(3.4)	-
	Metformin+glybiinclamid	0(0.0)	15(51.7)	-
	Metformin+glybiinclamid+ gliclazid	0(0.0)	3(10.3)	-

**Note:** Data are expressed as number (percentage). Percentages have been rounded and may not total 100. Analysis was Chi square or fisher exact test.

**Table 4:** Comparison of stage of change in oral anti-diabetic drugs (OADs) during the study.

Time	**SOC**	**Group**		*p*-Value^a^
		**Intervention Group (n = 32)**	**Non-Interventional Group (n = 29)**	
Baseline	Pre-Contemplation	5(15.6)	2(6.9)	.603
	Contemplation	1(3.1)	0(0.0)	
	Preparation	2(6.3)	3(10.3)	
	Action	3(9.4)	4(13.8)	
	Maintenance	21(65.6)	20(69.0)	
Post-intervention	Pre-Contemplation	0(0.0)	3(11.5)	.927
	Contemplation	0(0.0)	1(3.8)	
	Preparation	3(9.4)	4(15.4)	
	Action	10(31.3)	0(0.0)	
	Maintenance	19(59.4)	18(69.2)	
Follow up	Pre-Contemplation	2(6.3)	6(22.2)	.046
	Contemplation	0(0.0)	0(0.0)	
	Preparation	1(3.1)	3(11.1)	
	Action	2(6.3)	1(3.7)	
	Maintenance	27(84.4)	17(63.0)	
^b^*p*-value		.017	.575	

**Note:** Data are expressed as number (percentage). Percentages have been rounded and may not total 100. Analyses were ^a^ Mann Whitney, and ^b^ Friedman test. SOC: stages of change.

## Data Availability

The data supporting the findings of this study will be available from the corresponding author upon reasonable request.

## LIST OF ABBREVIATIONS

OADs: Oral Antidiabetic Drugs
T2DM: Type 2 Diabetes Mellitus
SOC: Stages of Change
FPG: Fasting Plasma Glucose
2-hPG: 2-hour Plasma Glucose
HOMA-IR: Homeostasis Model of Insulin Resistance Assessment
TTM: Trans Theoretical Model
RCT: Randomized Controlled Trial
RAS: Random Allocation Software
QSOCSMD-I: Questionnaire on the Stage of Change in the Self-management of Patients with T2DM, Iran
GLP-1: Glucagon-Like Peptide-1
DPP4: Dipeptidyl Peptidase 4

## STANDARDS OF REPORTING

CONSORT guidelines were followed.

## References

[1] Chakraborty S., Verma A., Garg R., Singh J., Verma H. (2023). Cardiometabolic risk factors associated with type 2 diabetes mellitus: A mechanistic insight.. Clin. Med. Insights Endocrinol. Diabetes.

[2] Yi Q.H., Wang H.L., Hou Y., Xu L., Tian W.L., Zhang Y.X., Xu Y.S., Shi J.B. (2024). A comparison of the decline in glomerular filtration rate between elderly patients with diabetes and those without diabetes in southwest China.. Endocr. Metab. Immune Disord. Drug Targets.

[3] Linkeviciute-Ulinskiene D., Kaceniene A., Dulskas A., Patasius A., Zabuliene L., Smailyte G. (2020). Increased mortality risk in people with type 2 diabetes mellitus in Lithuania.. Int. J. Environ. Res. Public Health.

[4] Kononenko IV, Smirnova OM, Mayorov AY, Shestakova MV (2020). Classification of diabetes. World health organization 2019. What’s new?. Diab. Mellit..

[5] Vishakh R., S. Kumari N., Bhandary A., Shetty S.S., Bhandary P., T. Selvan G. (2023). Evaluating the role of cytokinesis-block micronucleus assay as a biomarker for oxidative stress-inducing DNA damage in type 2 diabetes mellitus patients.. Beni. Suef Univ. J. Basic Appl. Sci..

[6] Akbarzadeh A., Salehi A., M. Vardanjani H., Poustchi H., Gandomkar A., Fattahi M.R., Malekzadeh R. (2019). Epidemiology of adult diabetes mellitus and its correlates in pars cohort study in Southern Iran.. Arch. Iran Med..

[7] Hu H., Sun W., Yu S., Hong X., Qian W., Tang B., Wang D., Yang L., Wang J., Mao C., Zhou L., Yuan G. (2014). Increased circulating levels of betatrophin in newly diagnosed type 2 diabetic patients.. Diabetes Care.

[8] Dilworth L., Facey A., Omoruyi F. (2021). Diabetes mellitus and its metabolic complications: The role of adipose tissues.. Int. J. Mol. Sci..

[9] Baek J.H., Kim H., Kim K.Y., Jung J. (2018). Insulin resistance and the risk of diabetes and dysglycemia in Korean general adult population.. Diabetes Metab. J..

[10] Park S.Y., Gautier J.F., Chon S. (2021). Assessment of insulin secretion and insulin resistance in human.. Diabetes Metab. J..

[11] Shah J. (2021). Insulin resistance and homeostatic model assessment in critically Ill: Where do we stand?. Indian J. Crit. Care Med..

[12] Marušić S., Meliš P., Lucijanić M., Grgurević I., Turčić P., Obreli Neto P.R., Bilić-Ćurčić I. (2018). Impact of pharmacotherapeutic education on medication adherence and adverse outcomes in patients with type 2 diabetes mellitus: A prospective, randomized study.. Croat. Med. J..

[13] Sugandh F.N.U., Chandio M., Raveena F.N.U., Kumar L., Karishma F.N.U., Khuwaja S., Memon U.A., Bai K., Kashif M., Varrassi G., Khatri M., Kumar S. (2023). Advances in the management of diabetes mellitus: A focus on personalized medicine.. Cureus.

[14] Natrus L., Lisakovska O., Smirnov A., Osadchuk Y., Savosko S., Klys Y. (2024). Could the propionic acid treatment in combination with metformin be safe for the small intestine of diabetic rats?. Endocr. Metab. Immune Disord. Drug Targets.

[15] Avramidis I., Apsemidou A., Lalia A.Z., Petridis N., Tourtouras E., Kalopitas G., Pilianidis G. (2020). Lessons from a diabetes clinic: Achieving glycemic goals and clinical use of antidiabetic agents in patients with type 2 diabetes.. Clin. Diabetes.

[16] Kakhki F.S.H., Asghari A., Bardaghi Z., Anaeigoudari A., Beheshti F., Salmani H., Hosseini M. (2024). The antidiabetic drug metformin attenuated depressive and anxiety-like behaviors and oxidative stress in the brain in a rodent model of inflammation induced by lipopolysaccharide in male rats.. Endocr. Metab. Immune Disord. Drug Targets.

[17] Krass I., Schieback P., Dhippayom T. (2015). Adherence to diabetes medication: A systematic review.. Diabet. Med..

[18] Rusdiana, Savira M., Amelia R. (2018). The effect of diabetes self-management education on Hba1c level and fasting blood sugar in type 2 diabetes mellitus patients in primary health care in Binjai City of North Sumatera, Indonesia.. Open Access Maced. J. Med. Sci..

[19] Gow K., Rashidi A., Whithead L. (2024). Factors influencing medication adherence among adults living with diabetes and comorbidities: A qualitative systematic review.. Curr. Diab. Rep..

[20] Du Pon E., El Azzati S., van Dooren A., Kleefstra N., Heerdink E., Van Dulmen S. (2019). Effects of a proactive interdisciplinary self-management (PRISMA) program on medication adherence in patients with type 2 diabetes in primary care: A randomized controlled trial.. Patient Prefer. Adherence.

[21] Selçuk-Tosun A., Zincir H. (2019). The effect on health outcomes of post-intervention transtheoretical model-based motivational interview in adults with type 2 diabetes mellitus: Follow up a cross-sectional study.. J. Caring Sci..

[22] Zare M., Tarighat-Esfanjani A., Rafraf M., Shaghaghi A., Asghari-Jafarabadi M., Shamshiri M. (2020). The barriers and facilitators of self-management among adults with type 2 diabetes mellitus: A trans theoretical model (TTM)-based mixed method study in Iran.. Diabetes Metab. Syndr. Obes..

[23] Krebs P., Norcross J.C., Nicholson J.M., Prochaska J.O. (2018). Stages of change and psychotherapy outcomes: A review and meta‐analysis.. J. Clin. Psychol..

[24] Zavarmoghadam M., Farmanbar R., M. zeidi I., Omidi S. (2018). The application of transtheoretical model to identify metabolic control and fruit and vegetable consumption in patients with type 2 diabetes.. Casp. J. Heal. Res..

[25] Centis E., Trento M., Dei Cas A., Pontiroli A.E., De Feo P., Bruno A., Sasdelli A.S., Arturi F., Strollo F., Vigili de’ Kreutzenberg S., Invitti C., Di Bonito P., Di Mauro M., Pugliese G., Molteni A., Marchesini G. (2014). Stage of change and motivation to healthy diet and habitual physical activity in type 2 diabetes.. Acta Diabetol..

[26] Arafat Y., M. Ibrahim M.I., Awaisu A. (2016). Using the transtheoretical model to enhance self-management activities in patients with type 2 diabetes: A systematic review.. J. Pharm. Health Serv. Res..

[27] Zare M., Rafraf M., Shaghaghi A., Jafarabadi M.A., Tarighat-Esfanjani A. (2020). Developing a valid and reliable questionnaire of the stages of change in self-management of patients with type 2 diabetes among an Iranian population.. Crescent J. Med. Biol. Sci..

[28] Chaiyasoot K., Sarasak R., Pheungruang B., Dawilai S., Pramyothin P., Boonyasiri A., Supapueng O., Jassil F.C., Yamwong P., Batterham R.L. (2018). Evaluation of a 12-week lifestyle education intervention with or without partial meal replacement in Thai adults with obesity and metabolic syndrome: A randomised trial.. Nutr. Diabetes.

[29] Basavareddy A., Dass A.S., Narayana S. (2015). Prediabetes awareness and practice among indian doctors: A cross-sectional study.. J. Clin. Diagn. Res..

[30] Folse H.J., Mukherjee J., Sheehan J.J., Ward A.J., Pelkey R.L., Dinh T.A., Qin L., Kim J. (2017). Delays in treatment intensification with oral antidiabetic drugs and risk of microvascular and macrovascular events in patients with poor glycaemic control: An individual patient simulation study.. Diabetes Obes. Metab..

[31] G. Moreno E., Mateo-Abad M., Ochoa de Retana García L., Vrotsou K., del Campo Pena E., Sánchez Perez Á., Martínez Carazo C., A. Ortiz J.C., Rúa Portu M.Á., Piñera Elorriaga K., Z. Pikatza A., U. Bengoa M.N., Méndez Sanpedro T., O. Portu A., A. Sorondo M.B., Rotaeche del Campo R. (2019). Efficacy of a self-management education programme on patients with type 2 diabetes in primary care: A randomised controlled trial.. Prim. Care Diabetes.

[32] Coppell K.J., Abel S.L., Freer T., Gray A., Sharp K., Norton J.K., Spedding T., Ward L., Whitehead L.C. (2017). The effectiveness of a primary care nursing-led dietary intervention for prediabetes: A mixed methods pilot study.. BMC Fam. Pract..

[33] Gathu C.W., Shabani J., Kunyiha N., Ratansi R. (2018). Effect of diabetes self-management education on glycaemic control among type 2 diabetic patients at a family medicine clinic in Kenya: A randomised controlled trial.. Afr. J. Prim. Health Care Fam. Med..

[34] Stubbs D.J., Levy N., Dhatariya K. (2017). Diabetes medication pharmacology.. BJA Educ..

[35] Powers M.A., Bardsley J., Cypress M., Duker P., Funnell M.M., Hess Fischl A., Maryniuk M.D., Siminerio L., Vivian E. (2015). Diabetes self-management education and support in type 2 diabetes: A joint position statement of the american diabetes association, the american association of diabetes educators, and the academy of nutrition and dietetics.. Diabetes Care.

[36] Liu X.L., Willis K., Wu C.J.J., Fulbrook P., Shi Y., Johnson M. (2019). Preparing chinese patients with comorbid heart disease and diabetes for home management: A mixed methods study.. BMJ Open.

[37] Zare M., Mardi A., Gaffari-moggadam M., Nezhad-dadgar N., Abazari M., Shadman A., Ziapour A. (2022). Reproductive health status of adolescent mothers in an Iranian setting: A cross-sectional study.. Reprod. Health.

[38] Zare M., Mardi A., Yeghanenia P., Hackett D. (2024). Healthy behaviors and gestational diabetes mellitus in an Iranian setting: A cross-sectional study.. Medicine.

[39] DiClemente C.C., Crisafulli M.A. (2022). Relapse on the road to recovery: Learning the lessons of failure on the way to successful behavior change.. J. Health Serv. Psychol..

[40] Norcross J.C., Krebs P.M., Prochaska J.O. (2011). Stages of change.. J. Clin. Psychol..

[41] Berry T., Naylor P.J., Wharf-Higgins J. (2005). Stages of change in adolescents: An examination of self-efficacy, decisional balance, and reasons for relapse.. J. Adolesc. Health.

