# Supplementary material for: Effect of a Medication Adherence Education Intervention on Clinical Outcomes in Adults with Type 2 Diabetes Mellitus: A Randomized Controlled Trial Study
Source: Endocr Metab Immune Disord Drug Targets. 2025 Jan 17;26:E18715303335172. doi: 10.2174/0118715303335172241216102015 (PMC13584076; doi:10.2174/0118715303335172241216102015)
Supplement: Supplementary file 1 [file EMIDDT-26-E18715303335172_SD1.pdf]

SUPPLEMENTARY MATERIAL

Effect of a Medication Adherence Education Intervention on Clinical Outcomes in Adults with Type 2 Diabetes Mellitus: A Randomized Controlled Trial Study

Ali Tarighat Esfanjani[1], Maryam Zare[2,*], Afrouz Mardi[3], Arezoo Haghighian Roudsari[4], Maryam Rafraf[5], Manouchehr Iranparvar-Alamdari[6,*], Daniel Hackett[7], Abdolreza Shaghaghi[8] and Mohammad Asghari Jafarabadi[9,10]

[1]Nutrition Research Center, Faculty of Nutrition and Food Sciences, Tabriz University of Medical Sciences, Tabriz, Iran; [2]Department of Nutrition, Khalkhal University of Medical Sciences, Khalkhal, Iran; [3]School of Health, Ardabil University of Medical Sciences, Ardebil, Iran; [4]Department of Community Nutrition, Faculty of Nutrition Sciences and Food Technology, National Nutrition and FoodTechnology Research Institute; Shahid Behshti University of Medical Sciences, Tehran, Iran; [5]Nutrition Research Center; Faculty of Nutrition and Food Sciences, Tabriz University of Medical Sciences, Tabriz, Iran; [6]Department of Internal Medicine, Faculty of Medicine, Ardabil University of Medical Science, Ardabil, Iran; [7]Physical Activity, Lifestyle, Ageing and Wellbeing Faculty Research Group, School of Health Sciences, Faculty of Medicine and Health, The University of Sydney, Lidcombe, NSW, Australia; [8]Department of Health Education and Promotion, Faculty of Health, Tabriz University of Medical Sciences, Tabriz, Iran; [9]Cabrini Research, Cabrini Health, VIC 3144, Malvern, Australia; School of Public Health and Preventive Medicine, Faculty of Medicine, Nursing and Health Sciences, Monash University, VIC 3800, Melbourne, Australia; [10]Road Traffic Injury Research Center, Faculty of Health, Tabriz University of Medical Sciences, Tabriz, Iran

BOX1. Session Box of oral anti-diabetic drugs (OADs) adherence education program.

SOC	Goal	Education Strategies
**Stage of change**	**Objective**	**trategies and learning objectivess Educational**
Pre-contemplation	To increase awareness of the need for taking OADs	Use of PowerPoint presentation and video projector to explain of complication of diabetes.<br><br>Encourage participants to think about complication of diabetes and related to do not taking OADs.<br><br>Provide information about the mechanisms of OADs.
Contemplation	To increase motivation of the need for promote self-management in field of taking OADs	Use of PowerPoint presentation and video projector to explain of the cognitive benefits of utilization self-management in field of taking OADs.<br><br>Improve subjects' self-efficacy to participate in this self-management.<br><br>Discuss the reasons with subjects for not taking OADs.<br><br>Explain the costs of complication diabetes treatment.<br><br>State the facilitating factors on conducting self-management in field of taking OADs.<br><br>Encourage participants to improve the understanding about importance o taking OADs.

Preparation	To negotiate a plan for Conducting self-management in field of taking OADs	Use of PowerPoint presentation to explain how to conduct OADs intervention.<br><br>Encourage participants to take action after they have learned some of self-management in field of taking OADs.<br><br>Plan some schemes with participants for conducting self-management in field of taking OADs.<br><br>Make a promise to subjects that they will conduct self-management in field of taking OADs and will improve the belief about behavior change.
Action and maintenance	To establish behavior habits and to review the Implementation of self-management in field of taking OADs	Ask participants about the methods of taking OADs.<br><br>Invite peers or family members about the implementation of participants' of self-management in field of taking OADs<br><br>Give the personalized feedback about self-management in field of taking OADs<br><br>Adjust the tendencies according to the conduction of the scheme.<br><br>Encourage participants to speak about the problems of that they encountered during conducting self-management in field of taking OADs<br><br>Participants will be aware of improvements function and motivate them to keep it up.<br><br>Integrate social support into the process of self-management in field of taking OADs.<br>Plan for resisting the temptation to skip of self-management in field of taking OADs.
